# Congenital urethral diverticulum with infertility in an adult man and review of the literature

**DOI:** 10.1186/s40064-016-3545-y

**Published:** 2016-10-24

**Authors:** Chen Shuzhu, Wu Min, Ye Weijing, Liu Yidong

**Affiliations:** Department of Urology, Ren Ji Hospital, School of Medicine, Shanghai Jiao Tong University, Shanghai, 200127 China

**Keywords:** Congenital, Urethral, Diverticulum, Ejaculation, Infertility

## Abstract

**Introduction:**

Congenital anterior urethral diverticula in adult males are infrequent urological diseases, which they were mainly found in women. The etiology of female diverticula is that (Mohan et al. in J Urol 123(4):592–594, 1980) women have anatomically poorly supported urethral. Clinical presentation frequently involves urinary urgency, polyuria, postmicturition dribble, and hematuria.

**Case description:**

A 37 year-old male was presented to us complaining of infertility about 6 years after marriage.

**Discussion and Evaluation:**

However, the complaint of infertility is extremely rare. Diagnostic imaging is useful to effectively confirm this disease in most cases. A complete review of the literature on this topic was also carried out.

**Conclusion:**

Manifestation as complaining of infertility is extremely rare among the congenital patients. The purpose of the operation is to complete the removal of the urethral diverticulum, reconstruct the urethra and maintain urinary tract unobstructed. This article and the operation could help the patient resolve the problem of infertility and dissatisfactory with the ejaculation.

## Case report

A 37 year-old male was presented to us complaining of infertility about 6 years after marriage in 2014.11. He also complained of unsatisfactory with ejaculation, dysuria, weak urinary stream, and significant post-micturition dribbling, without interruption of urinary stream and hematuresis. Swelling was a soft and fluctuant cystic which could be touched at the penis scrotum level. There is urine coming out when pressure being exerted on the cystic. Urine analysis, routine blood counts, blood urea, and serum creatinine are normal. Semen analysis, SCOU is normal.

**Fig. 1:**
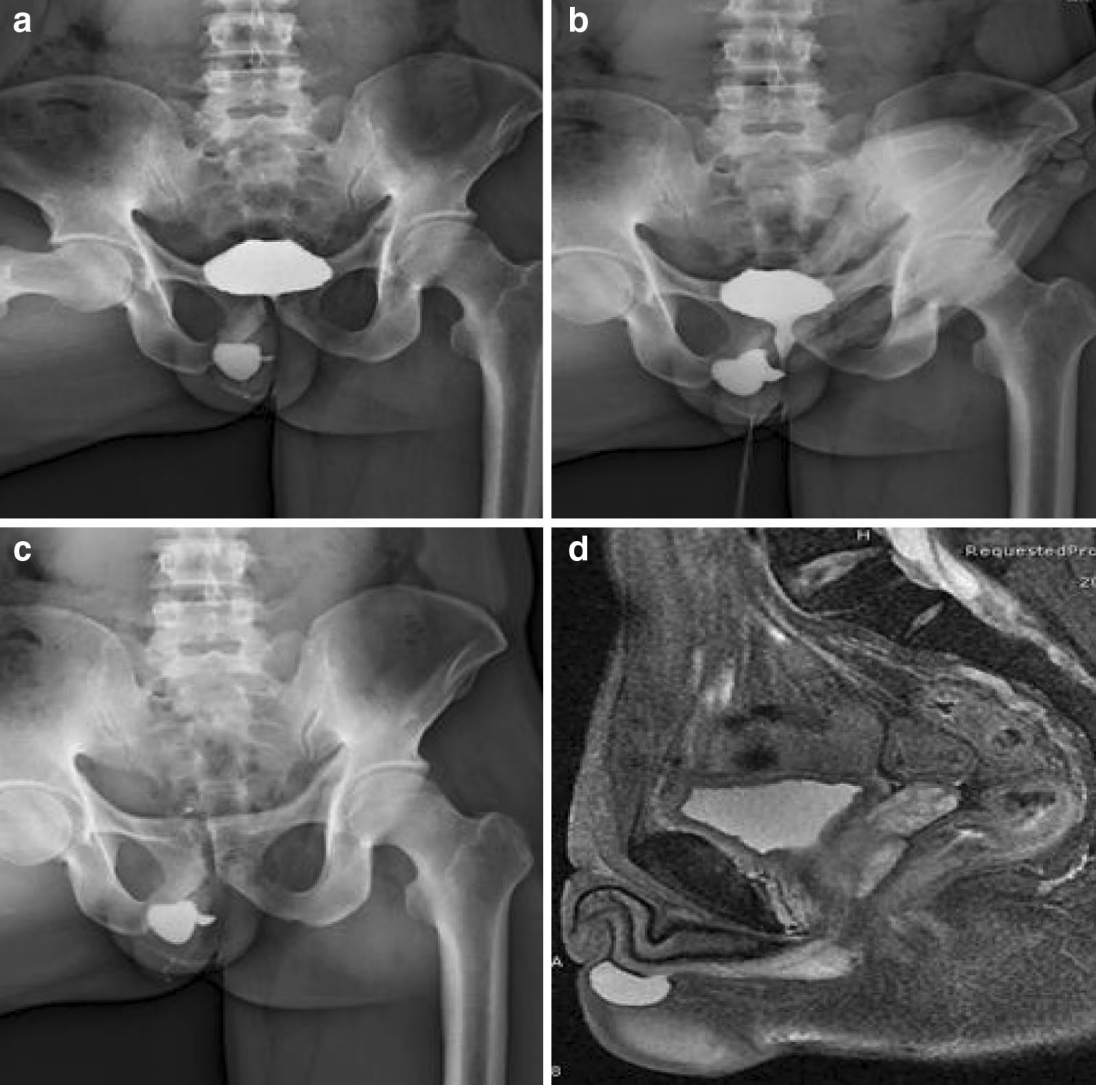
**a** Retrograde urethrogram showing the urethral diverticulum at the penoscrotal level (*A full bladder period*). **b** Retrograde urethrogram showing the urethral diverticulum at the penoscrotal level (*Emptying period*). **c** Retrograde urethrogram showing the urethral diverticulum at the penoscrotal level (*Empty period*). The swelling is still remain contrast filled after urethral emptying. **d** MRI reveals a fluid and hypodense mass correlated with urethra

**Fig. 2:**
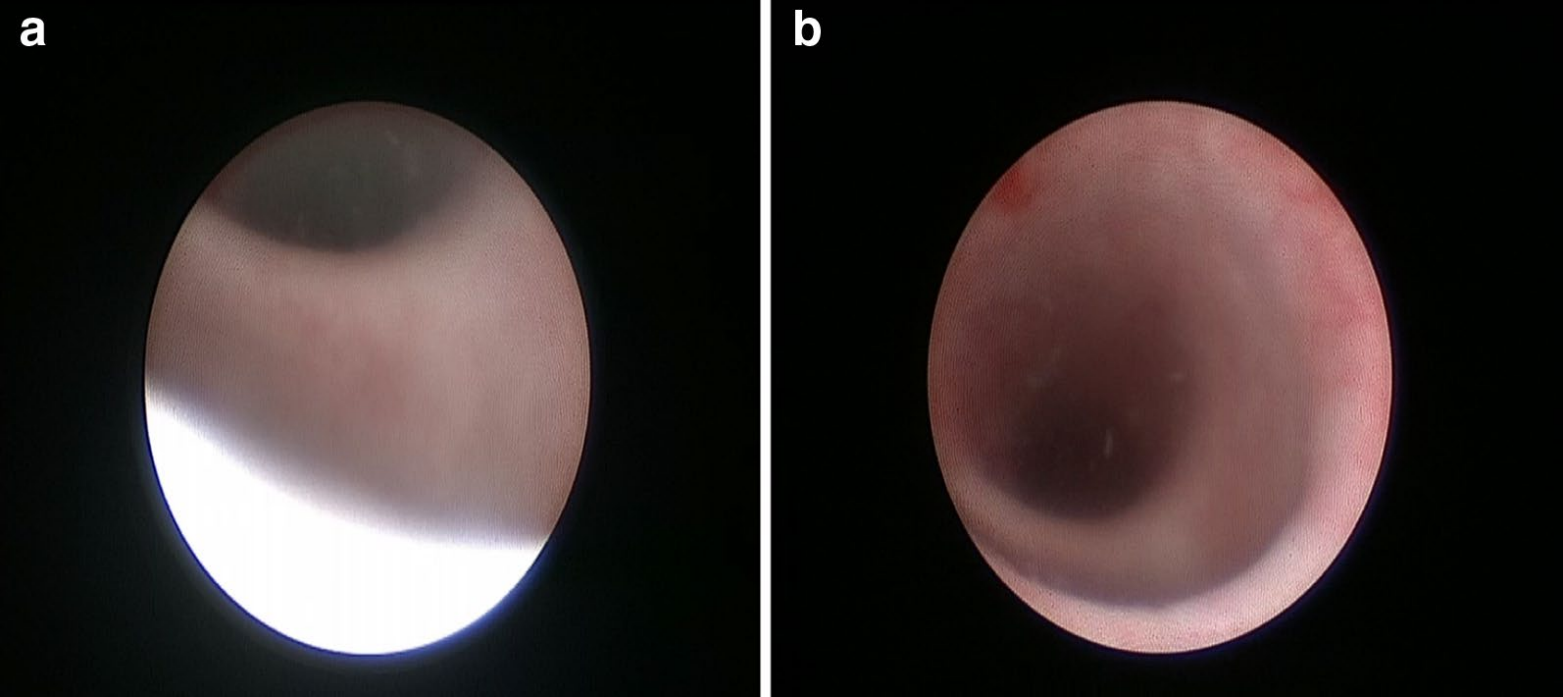
**a** Cystourethroscopy reveals a wide opening at the penoscrotal level. **b** Cystourethroscopy reveals a wide opening at the penoscrotal level, and the diverticulum has a smooth wall without any stones or focal infections

MRI revealing that there is a fluid and hypodense mass at the anterior corpus spongiosum which could not disappear after micturition (Fig. 1d). The mass is approximately 21 × 21 × 15 mm in diameter, which is correlated with urethra. It also presents a smooth boundary. The conclusion of MRI indicates that the patient has the possibility of having a urethral diverticulum. Ultrasonography showed kidney, bladder and testis normal in size and shape. Preoperative retrograde urethrogram (Fig. 1a,b,c) demonstrates that the patient has a cystic in the middle of urethral spongiosum. The contrast agents in the cystic did not disappear when the patient was asked to empty the urine. We could easily find the urethral diverticulum presented as penoscrotal swelling. The patient underwent a cystourethroscopy (Fig. 2a,b) which confirmed a large urethral diverticulum with a wide neck in the anterior. The cystic has a smooth boundary without any stones. We confirmed the diagnosis of the urethral diverticulum combined with clinical manifestations of the patient.

The patient was managed by open procedure. He received the urethral diverticulum resection and urethroplasty in the anesthesia. The basic operation procedure: The urethral diverticulum was opened by incision on the ventral aspects of the penis skin and fascia. Free urethral diverticulum from both sides completely, and give complete resection.

The post-operation pathological report indicates that it was lined by fibrovascular proliferation, granulation tissue and chronic inflammation, Post-operation recovery was uneventful. At 8 month follow-up, he had a normal urinary stream without any swelling, no urinary complaints, and he is satisfied with ejaculation. Of the most important, his wife was pregnant successfully. The report is according to the diagnosis of the urethral diverticulum.

## Discussion

Urethral diverticulum is divided into primary urethral diverticulum and acquired urethral diverticulum. Acquired diverticula as a consequence of urethral or meatal stenosis are a well-known complication of hypospadias operation (Ozgok et al. 1994). The other reasons we could easily find include inflammation, Trauma and tumor, as well as well as some secondary changes caused by urethroscope, catheter, and other medical instrument. The congenital diverticulum is rare in men, 90 % of which is secondary (Ortlip et al. 1980) and it is often appeared in female due to the reason that they have an anatomically poorly supported urethral. The men who could be diagnosed of the congenital diverticulum are mostly in childhood or adolescence, the average age of patients is 13 years old. Clinical manifestation of the urethral diverticulum included urinary infection, hematuresis, dysuresia, urine weakness and urinary tract obstruction.

The urethral diverticulum is defined (Mohan et al. 1980) as long tubular or banded structure whose openings are in the urinary tract. The most common localization is at the ventral aspect of the anterior bulbar urethra, and sometimes multiple diverticula are found. One classification system is based on the size of the diverticulum neck, which connects it to the urethra. Based on the width of the diverticulum (Rimon et al. 1992), the congenital urethral is divided into three kinds: wide-mouthed diverticulum, narrow-mouthed diverticulum and megalourethra (Ortlip et al. 1980; Monish et al. 1999).

Pathogenesis of congenital urethral: The etiology of the primary urethral diverticulum remains unclear, and several hypotheses have been reported to explain the etiology of the congenital urethral diverticulum. Among them, the following views led the trend (Gillitzer et al. 2008).

1. The defective closing of the bulbous portion of the urethra due to the partial lack of spongiosum tissue. It often occurs at the ventral aspect of the anterior urethra (Fahraeus 1991).
2. The occurrence of the urethral diverticulum which vanished later due to distal urethral valve obstruction (Sen et al. 1989).
3. Urethral diverticulum mostly occurs in the expansion of the cystic duct, Cooper’s gland or other urethral gland cystic (Fahraeus 1991).

Diagnosis basis: The initial diagnosis of a urethral diverticulum must be based on a high suspicion. Young men and children presenting with obstructive lower urinary tract symptoms and patients with risk factors for the development of acquired diverticula should be of caution. The male urethral diverticulum diagnosis mainly relies on the detailed medical history and related examinations, which include retrograde urography, IVU, MRI, Urethroscopy. Diagnosis rests essentially on the micturating cystourethrography (Paulhac et al. 2003). Retrograde urethrography may also suggest the presence of the anterior urethral diverticulum as in the present case. Magnetic resonance (MRI) is easy to operate, and it can show the size, shape, location of the urethral diverticulum more clearly, as well as the relationship with the peripheral tissue. MRI could also lead to fewer radiation injuries to the patient. So MRI is becoming more and more popular in imaging of the male urethral diverticulum. Urethroscope evaluation has the advantage of helping in visualization of associated anterior urethral valves, size of the neck of the diverticulum, appearance of the adjacent urethral mucosa and evaluation for feasibility of endoscopic definitive management. Ultrasound is used in partial clinic application. Ultrasound could inspect the penis or scrotal directly. The approach of ultrasonic examination includes by the perineum or the rectum is widely applied in clinical screening. In some cases, several examinations are needed for detecting the urethral diverticulum. But in some cases reported imaging studies are not always useful. Cai et al. (2008) reported one congenital case that imaging studies are not always useful for correct diagnosis, and that only a surgical approach can allow diagnosis of the presence of urethral diverticulum. This patient is the case that needs more examinations to make a definite diagnose due to the concealment of the illness.

The treatment principle: the treatment of the urethral diverticula should be according to own characteristic and the concomitant pathologic findings within the urethra. The post-operation pathological report of this case indicates that it was lined by fibrovascular proliferation, granulation tissue and chronic inflammation, so we could take measures relying on the following examination.

Small urethral diverticula with no symptoms could temporarily be neglected in therapy, and patients need to squeeze the urethral diverticulum under the guidance of the doctors to empty the urine staying in the urethral diverticulum after urinating. Some small diverticula needs endoscope considering of its atraumatic and simple characteristics. But In some cases, this method are not suitable due to the deficiencies of the surrounding supportive tissue and it could increase the formation of the fistula. So endoscopic treatment should be considered in caution. An open operation must be considered If endoscopic treatment is not appropriate. Our surgical goals (Rafique 2007) should include completely removing the diverticulum, keeping the continuity of the urethra, and also providing additional tissue to reinforce the repair. We should choose the simplest surgical option to achieve these goals and ensure that the repair has a good blood supply and under no tension. The alternative materials we choose could be the lingual tissue or buccal tissue.

**Fig. 3:**
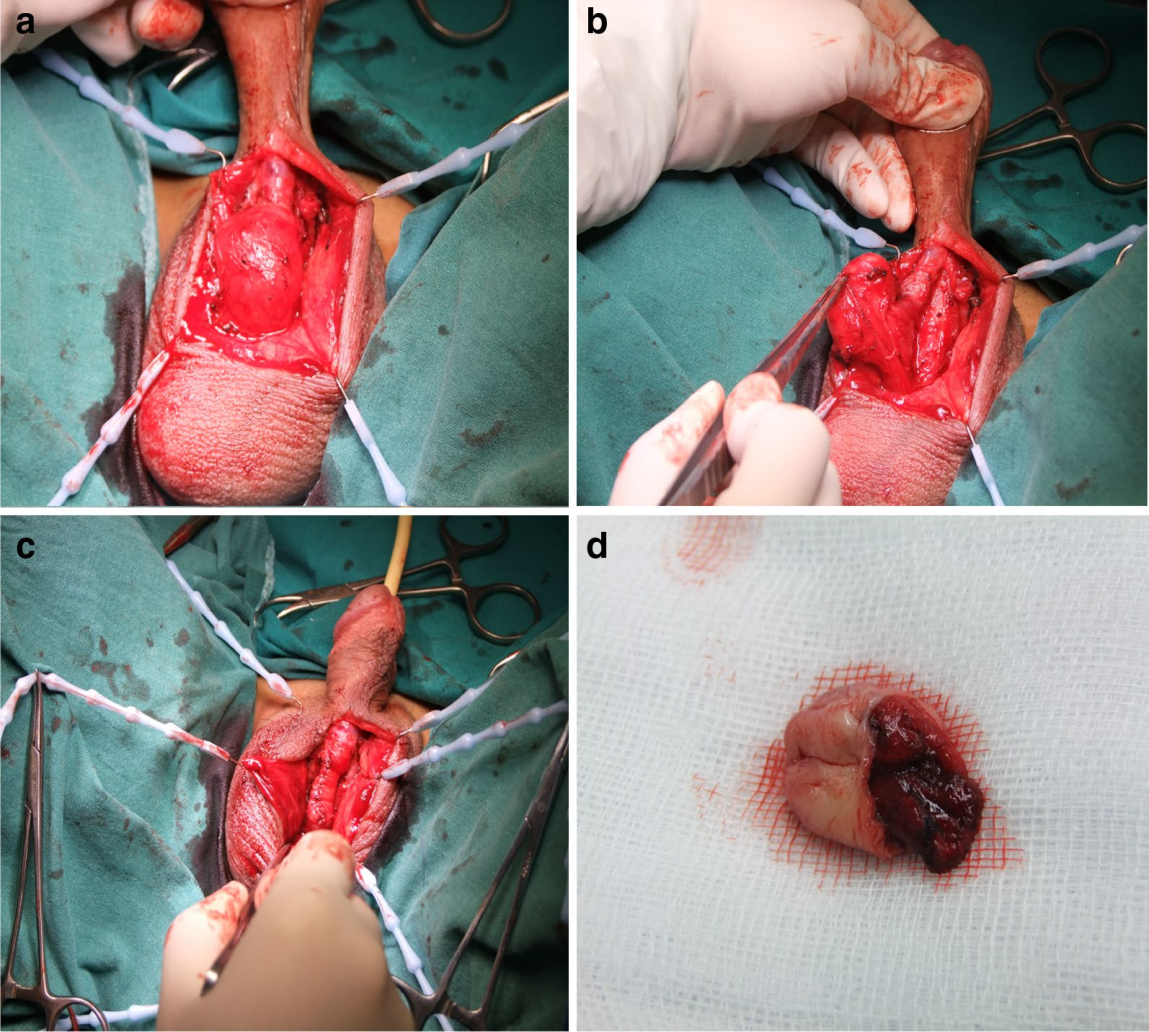
**a** The urethral diverticulum was opened by incision on ventral aspect of the penis skin and fascia, **b** free urethral diverticulum From both sides completely, and give complete resection. **c** Urethroplasty was performed following diverticulectomy **d** Diverticulum excised by operation

This urethral diverticulum of this patient is about 21 × 21 × 15 mm, large on volume. As the large urethral diverticulum has affected the patient’s life seriously, so we arrange an opening operation on the patient on the premise of conservative treatment being invalid. The urethral diverticulum is a mass which is lacked of sponge tissue. We need to remove about 3 cm. During the operation period, we should protect the sponge tissue of the urethra which is supposed to form the urethral and support the urethral wall. We make an incision on the ventral aspects of the penis skin and fascia (Fig. 3a). Free urethral diverticulum from both sides completely (Fig. 3b), and give complete resection (Fig. 3d). After removing the diverticulum, we finish suture (Fig. 3c). As the sponge tissue of this patient is rich after resection, we make an anatomy and provide additional sponge tissue to reinforce the repair and prevent a fistula to the skin. Post-operation recovery was uneventful. At 8 month follow-up, he had a normal urinary stream without any swelling, no urinary complaints, and he is satisfied with ejaculation. Of the most important, his wife was pregnant successfully. The report is according to the diagnosis of the urethral diverticulum.

## Conclusion

Manifestation as complaining of infertility is extremely rare among the congenital patients. The purpose of the operation is to complete the removal of the urethral diverticulum, reconstruct the urethra and maintain urinary tract unobstructed. This article and the operation could help the patient resolve the problem of infertility and dissatisfactory with the ejaculation.
